# Strategies to optimize stereotactic radiosurgery plans for brain tumors with volumetric‐modulated arc therapy

**DOI:** 10.1002/acm2.12818

**Published:** 2020-02-11

**Authors:** David Wang, Albert DeNittis, Yibing Hu

**Affiliations:** ^1^ Lankenau Medical Center Wynnewood PA USA; ^2^ Lankenau Institute for Medical Research Wynnewood PA USA

**Keywords:** brain metastasis, FI, HI, mGI, PCI, plan optimization, prescription isodose line, SRS, V12, VMAT

## Abstract

**Purpose:**

Prescription practice in SRS plans for brain tumors differs significantly for different modalities. In this retrospective study, the strategies to optimize SRS plans for brain tumors with volumetric arc therapy (VMAT) were presented.

**Methods:**

Fifty clinically treated cases were stratified by the maximum target size into two groups (≥ 2 cm in 25 cases and <2 cm but ≥1 cm in 25 cases), which were optimized using traditional LINAC MLC‐based approaches with the average prescription isodose line (P‐IDL) of (91.4 ± 0.6)%. Four to five plans have been created for each case with variation of the P‐IDL from 65% to 90%. The optimization strategies to select an optimal P‐IDL, to use tuning structures within the target and beyond as well as to use NTO (normal tissue objectives), were applied to all new plans.

**Results:**

The optimal P‐IDL was found to be around 75%. After applying the new optimization strategies with an average P‐IDL of 74.8%, the mean modified gradient index (mGI) and V12 were reduced by 25% and 35%, respectively for both groups. The Paddick conformity index (PCI) was averagely improved by 8%. The average homogeneity index (HI) and focal index (FI) were increased by 22% and 50%, respectively. The mGI was inversely proportional to the PTV volumes. The shape of the dose distribution in target was also changed from concave to convex. The comparison of PCI with historical data from other institutes and modalities shows that the plans in this study have the best conformity near the target.

**Conclusions:**

With the new optimization strategies for VMAT SRS plan of brain tumor more conformal plans in both high and intermediate dose region (~50% of the PD) were created, in which the dose in the core of the target was notably increased while V12 and mGI were significantly decreased, and PCI was improved. The mGI was inversely proportional to the PTV volumes. The optimal P‐IDL is around 75%. The average PCI is the best in this study compared with the published historical data. These strategies are applicable to treatment planning for multiple brain and liver tumors where sparing the tissue peripheral to the target is critical.

## 1 INTRODUCTION

SRS treatment for brain tumors requires conformity in both high and intermediate dose regions for better sparing normal brain tissue. The toxicities of SRS have been linked to V12, the volume of brain tissue irradiated with 12 Gy and conformity index.^1^, ^2^, ^3^ The dose prescription practice differs significantly among the modalities: Gamma Knife, Cyber Knife, and LINAC. The difference of the prescribed isodose line (P‐IDL, defined as the ratio of the prescription dose to the maximum dose in the target) can be as high as 40%.^4^, ^5^, ^6^

The SRS quality assurance and plan evaluation guidelines were proposed by the Radiation Therapy Oncology Group RTOG in the protocols 90–05 and 93–05,^7^, ^8^, ^9^ based on three parameters: HI (homogeneity index), defined by the ratio of the maximum dose in the target divided by the prescription dose (PD); CI (Conformity index), defined by the ratio of the prescription isodose surface volume divided by the target volume; and the appropriateness of target coverage, defined by minimum target dose divided by the prescription dose.^7^, ^8^, ^9^, ^10^ All three RTOG criteria stressed exclusively the coverage/sparing in the high dose region, with a wide range for each criterion.

A relatively large range of the RTOG criteria were proposed since all modalities were allowed. Traditionally the Gamma knife is a cone based device, prescribed to a low P‐IDL (~50%), whereas LINAC‐based systems are MLC based, can deliver dose with conformal static/arc/IMRT beams, prescribed to a higher P‐IDL (80–90%). For Cyberknife, the value of P‐IDL is often between those values.^11^ Previous studies^3^ showed that using either noncoplanar conformal static field or dynamic conformal arcs the optimal P‐IDL is around 77%. With development of volumetric arc therapy (VMAT) technique on LINAC, in which the beams are modulated for dose rate, MLC aperture and gantry angle continuously, more variables are introduced in the inverse planning and delivery process for SRS treatment.

The importance of dose fall outside of the tumor (intermediate dose region, i.e. 50% of prescription dose region) was omitted in the three RTOG criteria. The assumption implicitly meant that the smaller CI the more conformal of the plan, even though CI explicitly did not include any information for isodose volumes less than the prescription isodose volume. The definition of CI does not consider how much overlap of the volumes between the tumor and the volume covered by the prescription dose (PD). The assumption was incorrect when inverse optimization planning was implemented for IMRT and VMAT as so many variables were introduced. Clark et al^12^ proposed to implement the tuning structures outside of the target to aid in optimizing three dose level areas: high (~100% of PD), intermediate (~50% of PD), and lower dose spill (~40% of PD). Some tuning structures in target have been proposed by Soisson et al^13^ to improve the dose distribution in the target when planning SRS plans with helical tomotherapy. However the lack of overall optimization strategies, it was uncertain if the plans were optimal. We developed a set of systematic optimization strategies including using an optimal P‐IDL combining with implementing the tuning ring structures within the target and beyond, and implementing the normal tissue objectives. The new strategies were applied to the retrospective study of VMAT SRS plans for brain tumors including metastases and meningioma.

## 2 METHODS & MATERIALS

Fifty solitary brain tumors received single‐fraction cranial SRS from December 2014 to April 2017 in our department. The tumor diagnoses and characteristics are listed in Table 1. All patients were immobilized with the BrainLab frameless mask and fixation system (BrainLab, Feldkirchen, Germany). All treatment planning CT scans were performed in axial mode with a 1.25 mm slice thickness on a GE LightSpeed Scanner (GE healthcare, Chicago, USA). The CT scans have been fused with MRI images and the target was contoured based on MRI. Treatment plan system (TPS) was Eclipse V11.0 (Varian Medical Systems, Palo Alto, CA). All dose calculation was performed with AAA (Analytical Anisotropic Algorithm) and a resolution of 0.1 cm grid. All patients were treated with 10/6 MV FFF (flattening‐filter‐free) photon on a TrueBeam STx (Varian Medical Systems, Palo Alto, CA) equipped with ExacTrac kV imaging system (BrainLab, Feldkirchen, Germany), which allows verifying and monitoring patient position during treatment. The treatment couch top is a 6D movable device.

**Table 1:** Tumor diagnosis and characteristics are listed for a total 50 cases.

Diagnosis	Number of cases
Metastases	45
Meningioma	5
Maximum tumor size	
≥2 cm (Group Large)	25
<2 cm but ≥ 1 cm (Group Small)	25
Location of the tumor	
Peripheral	42
OARs within 2 cm but ≥ 1 cm	8

The beam isocenter in the treated plans was positioned automatically by the TPS at the center of the tumor. The collimator angle was determined so that the movement direction of HDMLC (high definition MLC) aligning with the minimum dimension of the target for best conformal plan.^14^ The treated plans were created with the intention to have more uniform dose in the target and the conventional P‐IDL (averagely (91.7 ± 0.5, ±1.7)% of the maximum dose. Results in the tables and text are reported as mean ± 95% confidence intervals, and ± STD.) was applied. All treated plans were 3 or 4 non‐coplanar partial RapicArcs and clinically accepted and delivered.

New optimization strategies for the retrospective study plans were compared with the old ones in Table 2 while keeping the same geometry of the plan, that is, the same number of arcs and range of arcs. The new optimization strategies have three crucial components^15^: 1. Adding the tuning ring structures in the target intends to make a convex dose distribution; 2. Implementing the NTO (normal tissue objectives) improves the conformity in the high dose region; 3. Adding the tuning ring structures outside the target makes the plan more conformal in the intermediate dose region. Combining with the selection of an optimal P‐IDL, more conformal plan in both high and intermediate dose regions with VMAT FFF were created. The tuning structures were created as concentric volumetric rings or enclosures with various radii within the target and beyond as follows:

- Within the target: three structures were created: R1, R2 and CTVCore, where R1 = PTV (planning target volume)‐CTV (clinical target volume) and R2 = CTV‐CTVCore, PTV = CTV+ 1mm, CTVCore = CTV‐the shell of the CTV with the thickness of a quarter of minimum dimension across the geometry center of the GTV (gross target volume).
- Beyond the target: Four structures were created: 2 mm, 5 mm, 2 cm and PartialBody, where 2cm = PartialBody‐(PTV + 2 cm), 8mm = PartialBody‐(PTV + 8 mm), 5 mm = PartialBody‐(PTV + 5 mm), the PartialBody is the part of the body in the volume of the PTV + 5 cm superiorly and inferiorly.

**Table 2:** Comparison of the two optimization approaches.

Optimization Strategies	Treated Plans	Retrospective study plans
Tuning ring structures within the target and beyond	Tuning ring structures outside of the target ONLY	Yes.
P‐IDL	(91.7 ± 0.5, ±1.7)%	(74.8 ± 0.6, ±1.7)%
Use NTO in the inverse plan optimization	No	Yes

NTO, normal tissue objectives.

**Figure 1:**
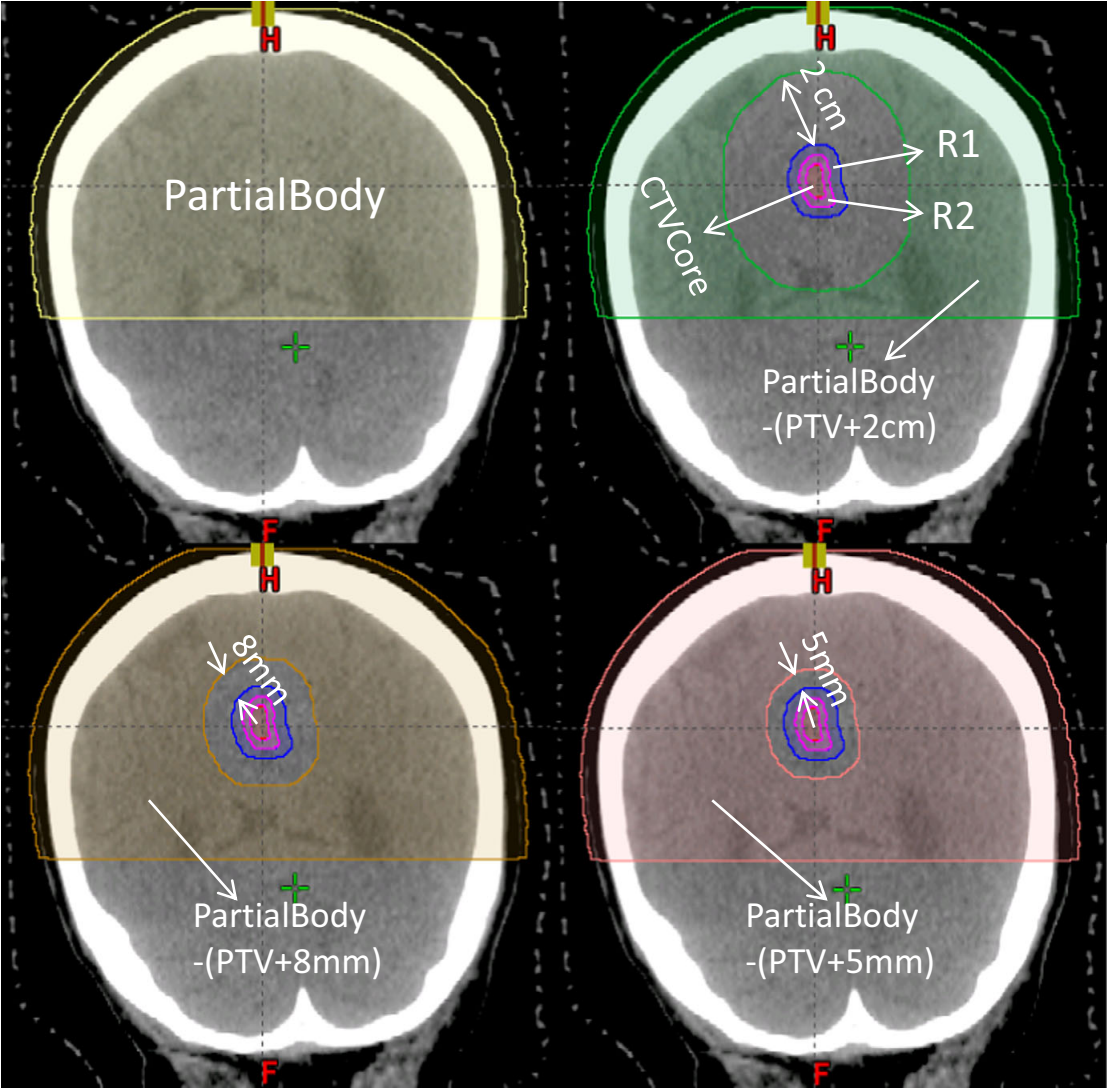
Illustration of the tuning ring structures. The partial body in the figure at the left up corner shows the contour of the partial body and aims at speed up optimization process due to reduced volume. The structures, PartialBody‐(PTV + 2 cm) in green, R1 in blue, R2 in pink and CTVCore in red, are shown at the top right of the Figure. The structures, PartialBody‐(PTV + 8 mm) and PartialBody‐(PTV + 5 mm), are shown at the bottom left and right of the figure, respectively. One can see that the three structures, PartialBody‐(PTV + 2 cm), PartialBody‐(PTV + 8 mm), and PartialBody‐(PTV + 5 mm), are partially overlapped.

The tuning structures are illustrated in Fig. 1. The initial settings of the objectives for the tuning ring structures present in Table 3. The NTO settings were listed as follows:

- Priority: 150
- Distance from target border: 0.03
- Start dose (%): 95
- End dose (%): 40
- Fall‐off: 0.8

**Table 3:** Settings for the tuning ring structures. Note that the dose percentage in the table is relative to the PD.

Structure	Upper			Lower			Lower		
	Vol (%)	Dose	Priority	Vol (%)	Dose	Priority	Vol (%)	Dose	Priority
R1	0	102.5%	150	100	97.5%	170			
R2	0	110%	150	100	102.5%	150			
CTVCore	0	120%	150	100	110%	150	50	115%	150
2 cm	0	2.5%	150						
8 mm	0	10%	150						
5 mm	0	20%	150						

PD, prescription dose.

To find out the optimal P‐IDL multiple plans with variation of P‐IDL from 65% to 90% have been created for each case. The variation in the P‐IDL with the following five indexes were recorded: Paddick comformity index,^16^ PCI (*PCI = TV_PTV_^2^/TV/PTV*), where TV_PTV_ is the volume of the target covered by the PD, PTV, the plan target volume and TV, the volume covered by the PD; *mGI* (Modified Gradient Index), the ratio of the volume covered by the 50% PD to the volume of the PTV covered by the PD; *V12*, the normal tissue volume irradiated within 12 Gy; Homogeneity Index (*HI*), the ratio of maximum dose to the PD. A new index, the Focal Index (*FI*), is proposed to represent the dose concentration in the target core, defined as D_min_·D_max_/PD,^2^ where D_min_ and D_max_ is the minimum and maximum dose in the target core region, respectively. The target core region is the volume of PTV minus the shell with thickness of 1/4 of the minimum dimension cross the geometry center of the PTV. Compared with the HI, the dose in the core region of the PTV can be better represented with the FI as it indicates a range of the dose not a point dose. After obtained the optimal P‐IDL, the retrospective study for the fifty plans was performed with the new optimization strategies while keeping the same arc arrangement and isocenter as the delivered plan. The coverage in all the plans was V_100%PD_ ≥ 98% PTV and Dmin. ≥95% PD.

## 3 RESULTS

**Figure 2:**
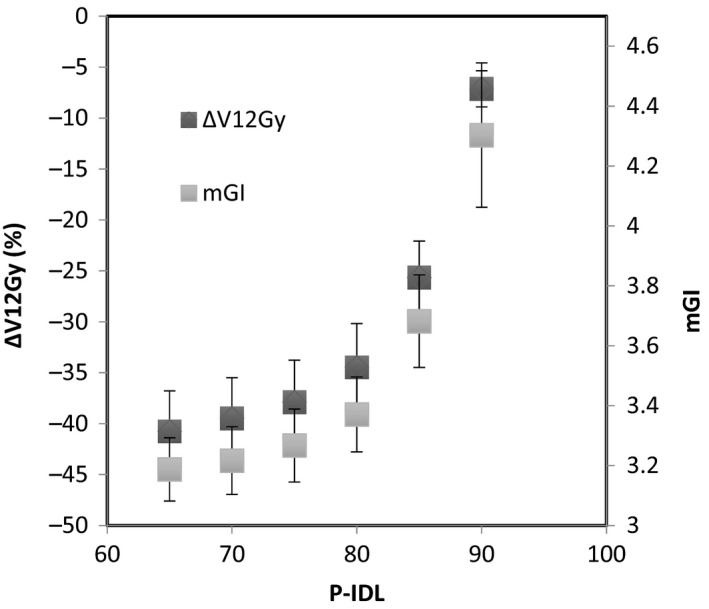
Variation of mGI and the relative change of V12 with the P‐IDL.

**Figure 3:**
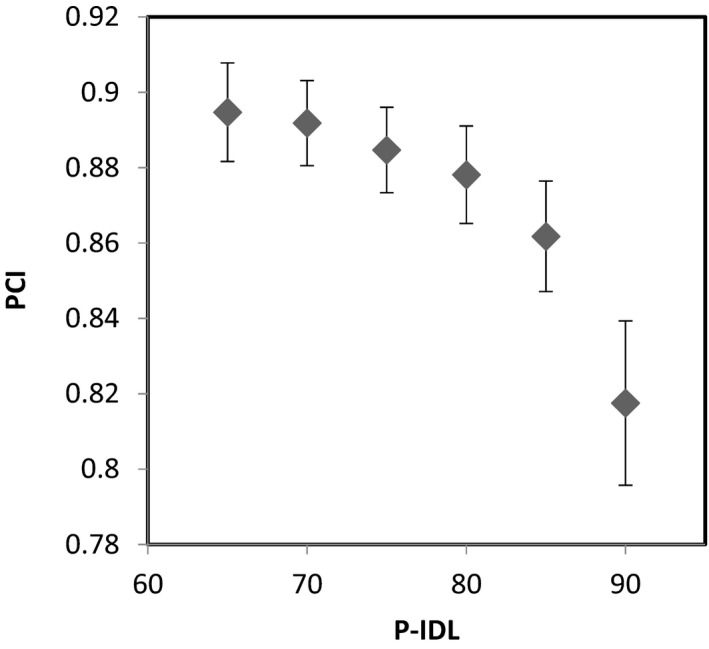
Variation of PCI with the P‐IDL. PCI, Paddick conformity index.

**Figure 4:**
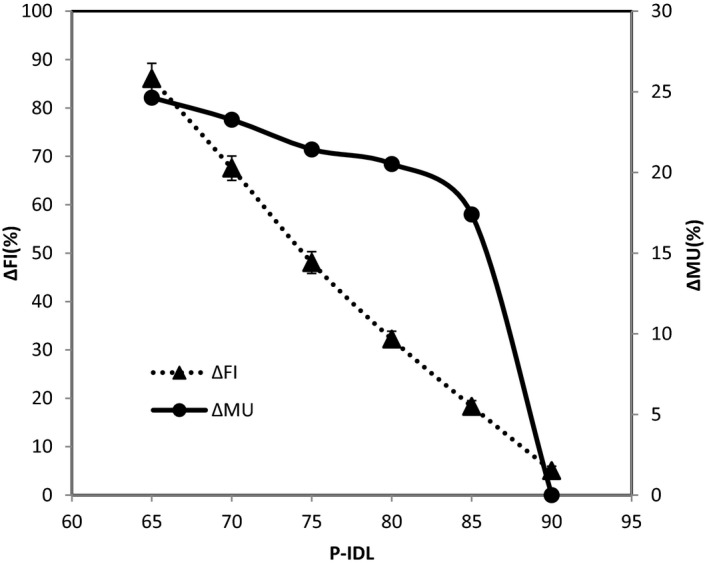
Variation of the relative change of FI and MU with P‐IDL.

The variation of mGI, PCI, and V12 with the P‐IDL from 65% to 90% for all the cases, in which the new optimization strategies were applied, are shown in Figs. 2, 3, 4, respectively. It can be seen that the change in mGI, PCI, and V12 with P‐IDL is relatively small beyond around 75% of P‐IDL (Fig. 2 and Fig. 3). Decreasing the P‐IDL further from 75%, the number of MU would increase more with the P‐IDL (Fig. 4) while other parameters would not improve significantly. Therefore overall the optimal range of the P‐IDL was determined to be at around 75%, which is comparable with that for conformal static field or dynamic conformal arcs.^3^ The HI and FI were inversely linearly proportional to the P‐IDL.

Comparison of the five parameters (mGI, V12, PCI, HI, and FI) for plan quality evaluation between the treated and retrospective study plans is presented in Table 4, in which the fifty cases were stratified into two groups with the maximum tumor size: ≥2 cm (Group Large), and < 2 cm but ≥ 1 cm (Group Small). The average P‐IDL of the retrospective study was (74.8 ± 0.6, ±1.7 STD)% while the average P‐IDL of the treated plans was (91.4 ± 0.6, ±1.7 STD)%.

**Table 4:** Comparison of the five criteria for plan quality evaluation between the treated and retrospective study plans.

Evaluation parameters		Treated Plans	Retrospective Study Plans	Difference (%)
mGI	Group Large	4.2 ± 0.3	3.1 ± 0.1	−26.0 ± 5.1
	Group Small	5.2 ± 0.4	3.9 ± 0.2	−24.5 ± 2.9
V12	Group Large	15.8 ± 3.9	9.5 ± 1.9	−39.9 ± 5.9
	Group Small	4.0 ± 0.5	2.6 ± 0.4	−35.0 ± 3.8
PCI	Group Large	0.83 ± 0.03	0.89 ± 0.01	7.2 ± 4.4
	Group Small	0.79 ± 0.02	0.88 ± 0.02	11.4 ± 4.2
HI	Group Large	1.08 ± 0.01	1.34 ± 0.01	23.4 ± 1.7
	Group Small	1.09 ± 0.01	1.34 ± 0.01	22.2 ± 1.3
FI	Group Large	1.07 ± 0.01	1.57 ± 0.04	47.1 ± 4.0
	Group Small	1.07 ± 0.01	1.60 ± 0.03	49.4 ± 3.1

PCI, Paddick conformity index.

**Figure 5:**
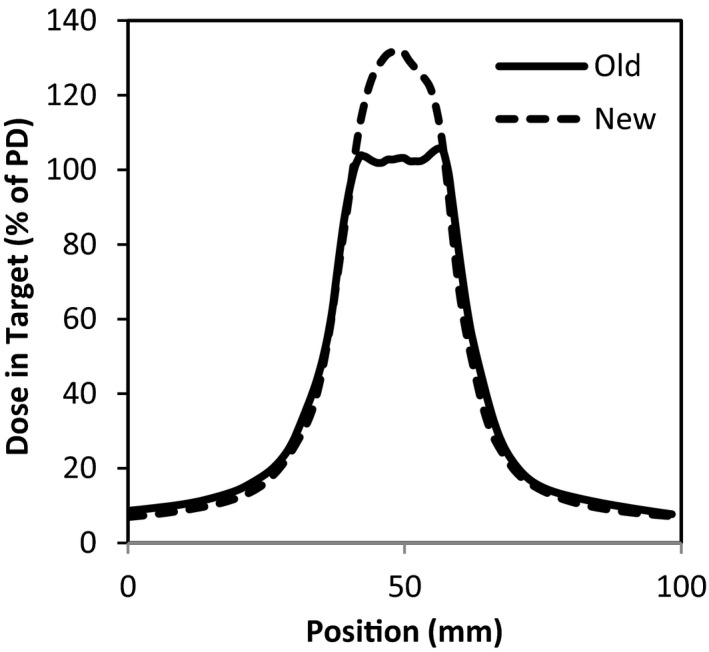
Comparison of typical dose distributions in the target for the treated plan (Old, solid line) and the retrospective study plan (New, dotted line).

**Figure 6:**
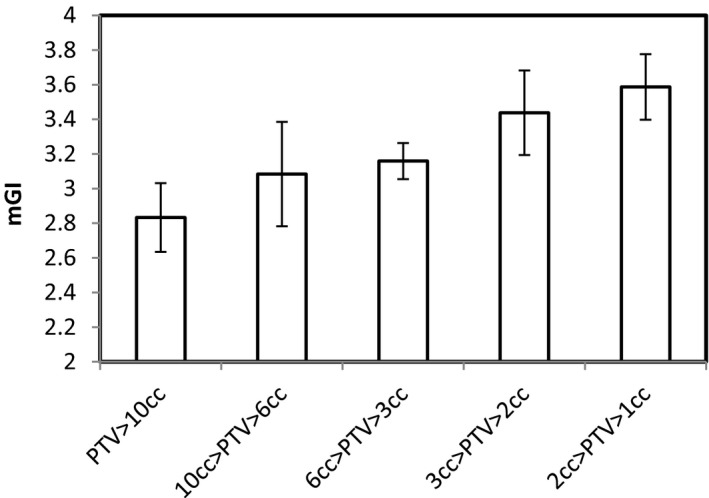
mGI was categorized into six groups according the PTV volumes for the retrospective study plans.

The average mGI was 4.2 ± 0.3 (±0.8 STD) and 5.2 ± 0.4, (±1.0 STD) for treated plan, while it was 3.1 ± 0.1, (±0.2 std) and 3.9 ± 0.2, (±0.5 std) for the retrospective study plans in the group large and small respectively. The mean mGI was reduced by about 25%. Although the V12 values depend on the PD, the reduction of the V12 was significant (more than 35% reduction for both groups) after implementing the new optimization strategies. The PCI was larger for the group small and improved by 11%. The average HIs for both groups were almost the same and were increased by 22% with the new optimization strategies. The FIs were increased almost 50% after implementing the new optimization strategies, that is, more dose has been delivered to the core of the PTV. A comparison of typical dose distributions in PTV between in the treated plans and in the retrospective study plan is shown in Fig. 5. It can be seen that it was a concave shape in the treated plan and a convex shape in the retrospective study plan. The mGI was inversely proportional to PTV volumes as shown in Fig. 6.

## 4 DISCUSSION

For dynamic conformal arc and conformal static treatment planning the P‐IDL is adjusted by changing the margin of the field and the dose distribution in the target is more or less inherently determined by the characteristics of the treatment unit. The optimal plan is optimized by a manually try‐and‐error process. However, VMAT optimization is an inverse process, i.e. the planner sets all parameters for a plan, let the TPS do the optimization. If the results are not clinically acceptable or optimal, one has to change the iteration and re‐optimize the plan till it meets certain criteria. The inverse optimization is an evaluation process based on the values of the cost functions for all the variables and if no variable is assigned to a dosimetric parameter, the optimization will ignore it. Without properly setting the iteration and knowing what crucial dosimetric parameters for plan evaluation, it would be difficult to create an optimal plan. Utilizing the tuning structures within the target and beyond in addition to the NTO, and selecting an optimal P‐IDL, forces the inverse planning system creating a more conformal plan in both the high dose and intermediate dose regions. The optimization process for multi‐targets in the VMAT planning for brain tumors is not different with that for single tumor except that collimator angle of the gantry might be crucial.^16^ Therefore, the new strategies are applicable to the optimization process of VMAT plan for multiple brain tumors.

**Figure 7:**
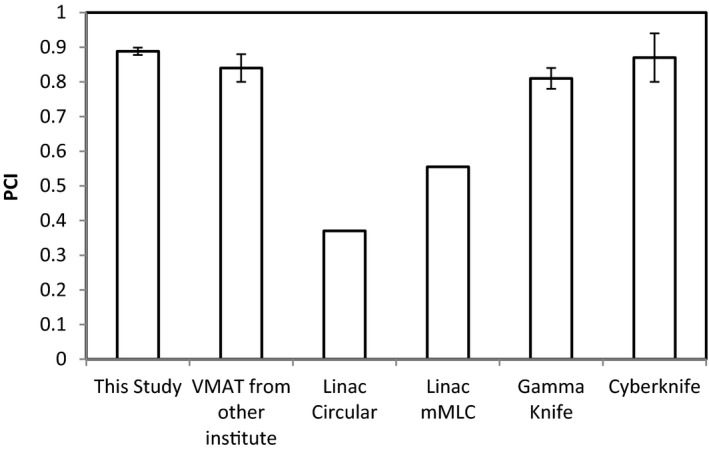
Comparison of PCI of this study to historical data. PCI, Paddick conformity index.

The PCI value varies little with the PTV size in this study, that is, 0.888 ± 0.01 although the PTV size varied from 1 to 16 cc. Fig. 7 compares the PCI of this study with the historical data from other institutes and modalities such as LINAC circular and mMLC,^18^ Cyberknife,^19^ Gamma Knife, and RapidArc.^20^

The V12, PCI, and mGI have been reported as key indicators for brain toxicities,^1^, ^2^, ^3^ such as radionecrosis, etc. The smaller the V12/mGI and the larger the PCI, the more is the brain tissue spared and the less the toxicities. Application of the new strategies has increased the core dose in the targets 50% as indicated in FI. In GammaKnife treated cases, in which there is no critical structures inside the target volume, overall there is no study demonstrating a relationship between the maximum dose in the target and complication rates.^16^ It was reported^9^ in the results of RTOG 9005 that patients treated on LINAC were 2.84 times more likely to have local progression compared to those treated on a Gamma Knife and the most significant difference of the treatment plans between the two modalities is the P‐IDL, which was 50% on a Gamma Knife and 80–90% on a LINAC. However this was not confirmed in the report of RTOG 9508.^17^ Further research is needed to see if there is correlation between local progression of brain metastases and the P‐IDL.

The factors to affect the P‐IDL probably are the penumbra of the MLC profiles, the scattering properties of the media (such as brain or lung, etc.) and the extent of modulation of the field. The penumbra of the MLC profiles is the inherent characteristics of a modality, which determines how steep of the dose falloff from the target. Modulation of fields like VMAT makes a plan more conformal by compensating the path length of beams in different directions if the tuning structures inside and outside of the target, and NTO are utilized in the inverse planning process.

## 5 CONCLUSIONS

With the new optimization strategies for VMAT SRS plan of brain tumor more conformal plans in both high and intermediate dose region (~50% of the PD) were created, in which the dose in the core of the target was notably increased while V12 and mGI were significantly decreased, and PCI was also improved. The optimal P‐IDL is around 75%. The average PCI is the best in this study compared with the published historical data. These strategies are applicable to treatment planning for multiple brain and liver tumors where sparing the tissue peripheral to the target is critical.

## CONFLICT OF INTEREST

There are no conflict of interests.
